# Supplementary material for: Allosteric regulation and crystallographic fragment screening of SARS-CoV-2 NSP15 endoribonuclease
Source: Nucleic Acids Res. 2023 Apr 28;51(10):5255–70. doi: 10.1093/nar/gkad314 (PMC10250223; doi:10.1093/nar/gkad314)
Supplement: gkad314_Supplemental_Files [file gkad314_supplemental_files.zip › Supplementary datasets.zip!/81/Refine_9/01-BUSTER/Cycle-2/shell.01/R_Rfree.html]

# Rxpct factors in resolution bins at cycle 0


|  |  |  |  |  |  |  |  |  |
| --- | --- | --- | --- | --- | --- | --- | --- | --- |
|  | **<d> (Å)**  Rxpct\_Work | Rxpct\_Free | Rxpct\_All | |  |  |  |  |
|  | 15.0454 | 0.2870 | 0.2208 | 0.2846 |  |
|  | 8.6865 | 0.1992 | 0.2413 | 0.2017 |  |
|  | 6.7285 | 0.2649 | 0.2343 | 0.2631 |  |
|  | 5.6866 | 0.2776 | 0.2670 | 0.2772 |  |
|  | 5.0151 | 0.2063 | 0.2043 | 0.2062 |  |
|  | 4.5364 | 0.1844 | 0.1893 | 0.1846 |  |
|  | 4.1728 | 0.2034 | 0.1775 | 0.2020 |  |
|  | 3.8847 | 0.2352 | 0.2293 | 0.2349 |  |
|  | 3.6491 | 0.2436 | 0.2361 | 0.2433 |  |
|  | 3.4517 | 0.2452 | 0.2245 | 0.2442 |  |
|  | 3.2832 | 0.2421 | 0.2553 | 0.2429 |  |
|  | 3.1372 | 0.2192 | 0.2259 | 0.2195 |  |
|  | 3.0091 | 0.2188 | 0.2227 | 0.2190 |  |
|  | 2.8955 | 0.2092 | 0.2055 | 0.2091 |  |
|  | 2.7939 | 0.2116 | 0.1855 | 0.2104 |  |
|  | 2.7022 | 0.2077 | 0.2234 | 0.2085 |  |
|  | 2.6191 | 0.2074 | 0.2327 | 0.2089 |  |
|  | 2.5431 | 0.2006 | 0.1977 | 0.2004 |  |
|  | 2.4735 | 0.1982 | 0.1916 | 0.1979 |  |
|  | 2.4092 | 0.2099 | 0.1899 | 0.2090 |  |
|  | **Overall** | 0.2254 | 0.2186 | 0.2250 |  |

# Rxpct factors in resolution bins at cycle 1


|  |  |  |  |  |  |  |  |  |
| --- | --- | --- | --- | --- | --- | --- | --- | --- |
|  | **<d> (Å)**  Rxpct\_Work | Rxpct\_Free | Rxpct\_All | |  |  |  |  |
|  | 15.0454 | 0.2863 | 0.2194 | 0.2838 |  |
|  | 8.6865 | 0.1996 | 0.2423 | 0.2021 |  |
|  | 6.7285 | 0.2651 | 0.2375 | 0.2635 |  |
|  | 5.6866 | 0.2776 | 0.2647 | 0.2772 |  |
|  | 5.0151 | 0.2069 | 0.2076 | 0.2069 |  |
|  | 4.5364 | 0.1857 | 0.1889 | 0.1859 |  |
|  | 4.1728 | 0.2051 | 0.1787 | 0.2037 |  |
|  | 3.8847 | 0.2360 | 0.2266 | 0.2356 |  |
|  | 3.6491 | 0.2448 | 0.2381 | 0.2445 |  |
|  | 3.4517 | 0.2477 | 0.2291 | 0.2468 |  |
|  | 3.2832 | 0.2466 | 0.2606 | 0.2475 |  |
|  | 3.1372 | 0.2261 | 0.2311 | 0.2263 |  |
|  | 3.0091 | 0.2250 | 0.2277 | 0.2251 |  |
|  | 2.8955 | 0.2151 | 0.2111 | 0.2149 |  |
|  | 2.7939 | 0.2138 | 0.1943 | 0.2129 |  |
|  | 2.7022 | 0.2076 | 0.2201 | 0.2083 |  |
|  | 2.6191 | 0.2043 | 0.2322 | 0.2060 |  |
|  | 2.5431 | 0.1995 | 0.1995 | 0.1995 |  |
|  | 2.4735 | 0.1962 | 0.1859 | 0.1957 |  |
|  | 2.4092 | 0.2083 | 0.1882 | 0.2074 |  |
|  | **Overall** | 0.2269 | 0.2203 | 0.2266 |  |

# Rxpct factors in resolution bins at cycle 2


|  |  |  |  |  |  |  |  |  |
| --- | --- | --- | --- | --- | --- | --- | --- | --- |
|  | **<d> (Å)**  Rxpct\_Work | Rxpct\_Free | Rxpct\_All | |  |  |  |  |
|  | 15.0454 | 0.2862 | 0.2189 | 0.2837 |  |
|  | 8.6865 | 0.1999 | 0.2430 | 0.2024 |  |
|  | 6.7285 | 0.2653 | 0.2398 | 0.2639 |  |
|  | 5.6866 | 0.2780 | 0.2631 | 0.2775 |  |
|  | 5.0151 | 0.2077 | 0.2104 | 0.2078 |  |
|  | 4.5364 | 0.1869 | 0.1892 | 0.1871 |  |
|  | 4.1728 | 0.2063 | 0.1799 | 0.2048 |  |
|  | 3.8847 | 0.2362 | 0.2258 | 0.2357 |  |
|  | 3.6491 | 0.2453 | 0.2386 | 0.2450 |  |
|  | 3.4517 | 0.2489 | 0.2314 | 0.2481 |  |
|  | 3.2832 | 0.2487 | 0.2622 | 0.2496 |  |
|  | 3.1372 | 0.2284 | 0.2344 | 0.2287 |  |
|  | 3.0091 | 0.2274 | 0.2297 | 0.2275 |  |
|  | 2.8955 | 0.2166 | 0.2134 | 0.2164 |  |
|  | 2.7939 | 0.2140 | 0.1949 | 0.2131 |  |
|  | 2.7022 | 0.2072 | 0.2201 | 0.2079 |  |
|  | 2.6191 | 0.2032 | 0.2308 | 0.2048 |  |
|  | 2.5431 | 0.1986 | 0.1980 | 0.1985 |  |
|  | 2.4735 | 0.1943 | 0.1822 | 0.1936 |  |
|  | 2.4092 | 0.2069 | 0.1868 | 0.2060 |  |
|  | **Overall** | 0.2276 | 0.2212 | 0.2273 |  |

# Rxpct factors in resolution bins at cycle 3


|  |  |  |  |  |  |  |  |  |
| --- | --- | --- | --- | --- | --- | --- | --- | --- |
|  | **<d> (Å)**  Rxpct\_Work | Rxpct\_Free | Rxpct\_All | |  |  |  |  |
|  | 15.0454 | 0.2936 | 0.2575 | 0.2923 |  |
|  | 8.6865 | 0.2323 | 0.2558 | 0.2337 |  |
|  | 6.7285 | 0.2938 | 0.2784 | 0.2929 |  |
|  | 5.6866 | 0.3243 | 0.3425 | 0.3249 |  |
|  | 5.0151 | 0.2567 | 0.2828 | 0.2584 |  |
|  | 4.5364 | 0.2380 | 0.2693 | 0.2395 |  |
|  | 4.1728 | 0.2465 | 0.2232 | 0.2452 |  |
|  | 3.8847 | 0.2677 | 0.2514 | 0.2669 |  |
|  | 3.6491 | 0.2753 | 0.2490 | 0.2740 |  |
|  | 3.4517 | 0.2796 | 0.2647 | 0.2788 |  |
|  | 3.2832 | 0.2815 | 0.3026 | 0.2828 |  |
|  | 3.1372 | 0.2699 | 0.2697 | 0.2699 |  |
|  | 3.0091 | 0.2580 | 0.2680 | 0.2584 |  |
|  | 2.8955 | 0.2420 | 0.2420 | 0.2420 |  |
|  | 2.7939 | 0.2255 | 0.2078 | 0.2246 |  |
|  | 2.7022 | 0.2071 | 0.2099 | 0.2073 |  |
|  | 2.6191 | 0.1862 | 0.2032 | 0.1872 |  |
|  | 2.5431 | 0.1771 | 0.1779 | 0.1771 |  |
|  | 2.4735 | 0.1672 | 0.1600 | 0.1669 |  |
|  | 2.4092 | 0.1728 | 0.1592 | 0.1722 |  |
|  | **Overall** | 0.2566 | 0.2555 | 0.2566 |  |
